# Risk Factors and Effects of Climate Lag on Vibrio Parahaemolyticus Infection in Eastern Coastal Cities of China: A Study Based on Hangzhou City

**DOI:** 10.3390/foods13132116

**Published:** 2024-07-02

**Authors:** Hangqi Ren, Ting Liu, Hao Hou, Xiaojuan Qi, Lei Fang, Yinyi Yang, Rong Ma

**Affiliations:** 1 Zhejiang Provincial Key Laboratory of Urban Wetlands and Regional Change, Hangzhou Normal University, Hangzhou 311121, China; 2021210214015@stu.hznu.edu.cn (H.R.); houhao@hznu.edu.cn (H.H.); 2021210214012@stu.hznu.edu.cn (R.M.); 2 Zhejiang Provincial Center for Disease Control and Prevention, Hangzhou 310051, China; xiqi@cdc.zj.cn; 3 Department of Environmental Science and Engineering, Fudan University, Shanghai 200438, China; fanglei@fudan.edu.cn; 4 Institute of Science, Technology and Society, South China Normal University, Guangzhou 510006, China; 2023025021@m.scnu.edu.cn

**Keywords:** *Vibrio parahaemolyticus*, impact factors, logistic regression, temperature lag

## Abstract

Bacterial foodborne diseases caused by *Vibrio parahaemolyticus* pose persistent challenges to coastal cities in China. In this study, we employed multiple logistic regression analysis and distributed lag non-linear models (DLNM) to investigate the epidemiological characteristics and associated risk factors of vibriosis in the metropolitan area of Hangzhou from 2014 to 2018. Analysis of foodborne cases indicated that certain demographics and occupational factors, including age between 16 and 44 years; houseworkers or unemployed individuals; preference for aquatic and meat products; and dining in collective canteens or catering services contribute to an increased likelihood of *V. parahaemolyticus* infection. Moreover, a higher per capita GDP and exposure to high temperatures were identified as risk factors for vibriosis. This study highlights the significance of the daily mean temperature as a meteorological factor influencing *V. parahaemolyticus* infection, with varying lag effects observed depending on temperature conditions. At low temperatures, the risk of infection occurs after a lag of 21 days, whereas at high temperatures, the risk is highest on the same day, while the second infection risk period occurs after a lag of 21 days. These findings provide a spatiotemporal perspective of the risk analysis of foodborne diseases, with a daily timescale and street spatial scale, which contributes to the development of public health strategies and food safety protocols in coastal cities.

## 1. Introduction

Foodborne illnesses are caused by the ingestion of contaminated food and food products, including those contaminated by pathogens, chemicals, and parasites during food production and preparation [1]. Globally, diarrhea alone caused more than 1.3 million deaths in 2015 and more than 1.6 million deaths by 2016 [2]. *Vibrio parahaemolyticus* has been a major cause of bacterial foodborne outbreaks in China over the past four decades [3,4], especially in coastal provinces [5]. Among the 13,307 foodborne disease outbreaks of known etiology that occurred in China from 2003 to 2017, *V. parahaemolyticus* accounted for 11.3%, ranking second among all diseases [6]. *V. parahaemolyticus* has also been a major cause of bacterial foodborne disease outbreaks in many Asian countries, including Japan and India [7,8,9]. The consumption of raw or undercooked fish and aquatic plants is a traditional dietary practice in these countries [10]. In general, the consumption of food contaminated with *V. parahaemolyticus* may lead to acute gastroenteritis, which clinically manifests as diarrhea, headache, vomiting, nausea, abdominal pain, and mild fever [8]. The epidemiological importance of *V. parahaemolyticus* foodborne illness is particularly important in urban areas because of their high population density, diverse food culture, and complex food supply chain. Therefore, understanding the factors that influence the pathogenesis of this disease is essential for safeguarding public health.

*V. parahaemolyticus* is a Gram-negative halophilic bacterium that is widely found in seawater, marine fish, shellfish, and shrimp in China [11]. Several studies [12,13,14] have examined *V. parahaemolyticus* in aquatic products, specifically focusing on the concentration and distribution of this bacterium under different environmental conditions. The lowest growth temperature of *V. parahaemolyticus* was 8.3 °C, and the optimum growth temperature was 37–39 °C [15]. The density of *V. parahaemolyticus* showed a strong seasonal trend, and its maximum density appeared in June, two months before the highest seasonal water temperature [16]. *V. parahaemolyticus* outbreaks are usually associated with contaminated or improperly cooked aquatic products. In China’s coastal cities, often eating out and eating raw (undercooked) seafood and meat can easily lead to *V. parahaemolyticus* infection [17,18].

Previous studies have analyzed the main influencing factors of local *V. parahaemolyticus* infection by examining the socioeconomic status [19], dietary history [20], and exposure environment of the infected cases [21]. According to survey analysis, people with higher socioeconomic status often exhibit higher food safety awareness and adhere to better hygiene habits [22]. However, this higher socioeconomic status can also lead to an increased likelihood of engaging in high-risk behaviors, such as dining out or consuming seafood [19]. This paradoxical relationship highlights the complexity of socioeconomic status as a contributing factor. Climatic factors also play a crucial role in *V. parahaemolyticus* infection. Climate change not only affects the growth of microorganisms, but also affects the physiology of animals and the susceptibility of hosts [23,24]. Consequently, this can lead to an increased risk of human infection [25,26,27]. Global warming has resulted in a gradual rise in ocean temperature, creating a more favorable environment for the growth of pathogens like *V. parahaemolyticus*. For example, extreme heat waves in the high latitudes of the northern hemisphere and ocean warming have been linked to an increase in vibrio infections [28,29]. Temperature has been identified as the climatic factor most strongly correlated with the infection rate of vibriosis [30]. *V. parahaemolyticus* can cause infection at lower temperatures, as seen in Alaskan waters where infection occurs at 15 °C [31] and then warm air temperatures promote rapid growth of the bacteria in seafood after harvest. For example, *V. parahaemolyticus* multiplies at an alarming rate in live oysters at 26 °C, increasing by 790 times in just 24 h [32]. These findings further emphasize the significant impact of temperature on the risk of *V. parahaemolyticus* infection.

In addition, there is a lag effect observed in the impact of climate factors, such as ambient temperature, on *V. parahaemolyticus* infection [33,34]. Higher temperatures do not immediately accelerate the growth and reproduction of *V. parahaemolyticus*, but they do result in shorter lag times and faster growth rates [35,36,37]. In Zhejiang Province, for example, there was a lag of approximately three weeks between temperature changes and *V. parahaemolyticus* infection cases [38]. Similarly, studies conducted in Taiwan have demonstrated a significant association between extremely high temperatures and an increased risk of bacterial diarrhea when lagged by 8 weeks [39]. However, the current research on the epidemiological characteristics of foodborne diseases primarily relies on monthly [40,41,42], or weekly [39] time scales, as well as spatial scales at the city or state level [43,44,45]. This research methodology may not fully capture the lag effect caused by the daily changes in climatic factors, and it can be challenging to identify subtle differences within geographical spaces within a city.

Hangzhou, situated in the eastern coastal region of China, is known for its prosperous economy and dense population, making it a high-risk area for *V. parahaemolyticus* infections. The objective of this study was to comprehensively understand the epidemiological characteristics and risk factors associated with vibriosis in Hangzhou. Daily foodborne disease data in the main urban area of Hangzhou were collected, and street-scale spatial data were integrated to analyze the influencing factors of vibriosis from three perspectives: population structure, socioeconomic characteristics, and meteorological environmental factors. In this study, we pay special attention to the effects of climatic factors on foodborne diseases of *V. parahaemolyticus* and analyze the immediate and delayed effects under different temperature scenarios. By doing so, this study aims to enhance our comprehensive understanding of the dynamic process of foodborne diseases.

## 2. Materials and Methods

### 2.1. Study Area

**Figure 1:**
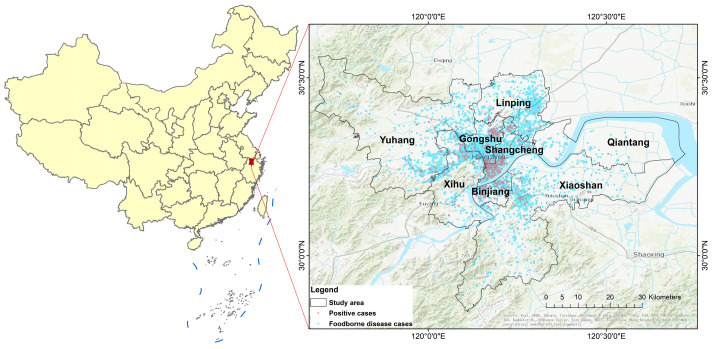
Distribution of foodborne disease cases in the main urban area of Hangzhou City.

In this study, we selected the urban area of Hangzhou (Urban District), with a relatively concentrated population, prosperous economy, and high urbanization rate, as the research area, including Shangcheng, Gongshu, Binjiang, Xihu, Linping, Qiantang, Xiaoshan, and Yuhang Districts. Between 2014 and 2018, 11,020 foodborne disease cases were diagnosed at 15 sentinel hospitals in the study area. We used the Gaode (AMap) Map geocoding API (https://restapi.amap.com/v3/geocode/geo, accessed on 12 March 2023) to obtain the spatial data of the street-scale cases. Using this geocoding API, the residential addresses and suspected exposed food-eating places of the cases were successfully converted into latitude and longitude coordinates, along with their corresponding matching levels obtained from the address geocoding process. We specifically retained the resolved results that exceeded the township matching level, thereby establishing a spatial scale at the street level for the cases’ spatial data. A case distribution map based on the spatial data of these cases is shown in Figure 1.

### 2.2. Data Source

**Figure 2:**
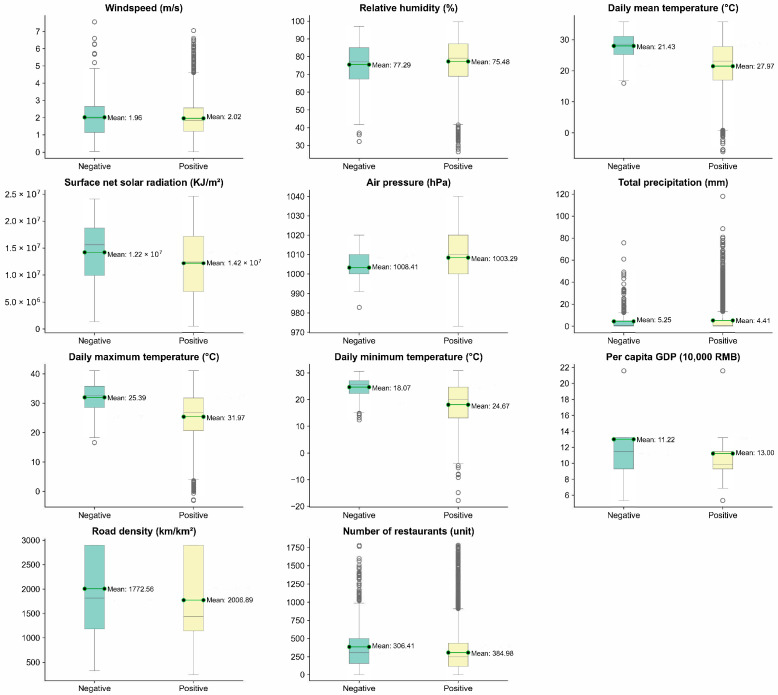
Distribution of social and meteorological data under *V. parahaemolyticus* detection results.

To study the factors influencing *V. parahaemolyticus* infection in the main urban area of Hangzhou City, we included data for three main aspects: reported cases from the foodborne disease surveillance system of Zhejiang Province, meteorological and environmental factors, and socioeconomic factors, as shown in Figure 2. Case data were obtained from the monitoring reports of 15 sentinel hospitals in the main urban area of Hangzhou from 2014 to 2018, including information on the patient’s sex, age, occupation, suspected exposed food, and the place where the suspected exposed food was eaten. Meteorological environmental factor data, spanning the years 2014 to 2018, were obtained from the ERA5-Land database (https://www.ecmwf.int/, accessed on 20 June 2023). The dataset includes variables such as wind speed (m/s), relative humidity (%), daily mean temperature (°C), net surface solar radiation (KJ/m2), air pressure (hPa), total precipitation (mm), daily maximum temperature (°C), and daily minimum temperature (°C). The per capita GDP data in the socioeconomic factor data were obtained from the Center for Resource and Environmental Science and Data of the Chinese Academy of Sciences (https://www.resdc.cn/, accessed on 28 July 2023). The road density data in the Hangzhou main urban area were obtained from the OpenStreetMap website (https://www.openstreetmap.org/, accessed on 23 May 2023), and the data on the number of food and beverage outlets within a 1 km radius in the vicinity were obtained from the POI data from the Amap POI search API. During data processing, we used spatial interpolation to geocorrelate meteorological and socioeconomic factors in each case.

### 2.3. Data Analysis

#### 2.3.1. Spatial Analysis and Epidemiological Statistics

We used general statistical descriptive methods to perform epidemiological characterization of the case data, including population age structure, occupational characteristics, food exposure, and temporal characteristics. The kernel density estimation (KDE) method [46,47] was used to spatially estimate the case data at the pixel scale, resulting in separate kernel density maps for all *V. parahaemolyticus* detection cases and cases with positive detections of *V. parahaemolyticus*. By dividing these two maps, we obtained a kernel density map of the *V. parahaemolyticus* detection rates in the main urban area of Hangzhou, which demonstrated the spatial distribution of the *V. parahaemolyticus* detection rates.

The calculation formula for kernel density estimation is as follows [48]:

(1) $$\hat{f}\left(x,y\right)=\frac{1}{{\mathrm{nh}}^{2}}\sum_{i=1}^{n}K(\frac{\sqrt{{(x-x_{i})}^{2}+(y-y_{i}{)}^{2}}}{h})$$

where $\hat{f}\left(x,y\right)$ is the estimated value of the kernel density at position $\left(x,y\right)$, n is the number of points in the two-dimensional spatial dataset, h is the bandwidth parameter, and $K\left(u\right)$ is the kernel function that represents the weight of the distance parameter *u* and is the coordinate of each point in the two-dimensional spatial dataset.

#### 2.3.2. Risk Factor Analysis

In this study, a multiple logistic regression model was used to explore the risk factors of *V. parahaemolyticus* infection from the perspectives of case characteristics, social economy, and meteorological environment. After removing irrelevant factors using the chi-square test and independent sample *t*-test, the model considered variables such as patient age, occupation, type of exposed food, eating place of exposed food, meteorological environmental factors, and socioeconomic factors. The age distribution of patients covered different age groups, including 0–15 years (3520/11,020), 16–24 years (1554/11,020), 25–44 years (3581/11,020), 45–64 years (1672/11,020), and ≥65 years (693/11,020). Occupations included students (4222/11,020), migrant workers (1332/11,020), cadres and staff (2627/11,020), houseworkers and the unemployed (1386/11,020), and other occupations (1453/11,020). The food categories included cereals products (1267/11,020), meat products (1758/11,020), vegetable products (993/11,020), aquatic products (1931/11,020), dairy products (876/11,020), and other categories (4195/11,020). The exposed food-eating places included retail markets (922/11,020), home (5646/11,020), collective canteens (174/11,020), catering services (934/11,020), and others (3344/11,020). Meteorological environmental factors such as relative humidity, daily mean temperature, surface net solar radiation, air pressure, total precipitation, daily maximum temperature, and daily minimum temperature were also considered, as were socioeconomic factors such as road density, per capita GDP, and the number of restaurants within a 1 km range. Some climatic variables were excluded due to their multicollinearity. For example, there is significant collinearity between the daily mean temperature and daily maximum and minimum temperatures, a high correlation between air pressure and temperature, and a high correlation between solar radiation and relative humidity.

#### 2.3.3. Distributed Lag Non-Linear Model

The distributed lag non-linear model (DLNM) has been widely used to assess the relationship between the lag effects of climate variables and infectious diseases [39,49]. In this study, the DLNM proposed by Gasparrini [50] was used to evaluate the delayed effects of climatic factors on foodborne diseases caused by *V. parahaemolyticus* in the main urban area of Hangzhou. In this study, we explained the effects of meteorological factors on *V. parahaemolyticus* diseases by fitting a dichotomous logistic regression model [51] as follows:

(2) $$\log\left(\frac{p_{i}}{1-p_{i}}\right){\;=\beta }_{0}{\;+\;\beta }_{1}\cdot \mathrm{basis}{.\mathrm{Tem}}_{i}{\;+\;\beta }_{2}\cdot \mathrm{ns}{\left(\mathrm{date},7\right)}_{i}{\;+\;\beta }_{3}\cdot {\mathrm{RH}+\beta }_{4}\cdot {\mathrm{RA}+\beta }_{5}\cdot {\mathrm{Density}+\beta }_{6}\cdot {\mathrm{GDP}+\beta }_{7}\cdot {\mathrm{Count}+\beta }_{8}\cdot \mathrm{DOW}$$

where $p_{i}$ is the probability of the occurrence of a specific foodborne illness for the *i*th observation; $\log\left(\frac{p_{i}}{1-p_{i}}\right)$ is the log odds ratio for the *i*th observation; $\beta_{0}{,\;\beta }_{1}{,\;\beta }_{2}{,\;\beta }_{3}{,\;\beta }_{4}{,\;\beta }_{5}{,\;\beta }_{6}{,\;\beta }_{7}{,\;\beta }_{8}$ are the model parameters, including the intercept and the coefficients of the respective variables; $basis.Tem_{i}$ represents the basis function transformation of the daily mean temperature for the *i*th observation; and *DOW* represents the day of the week effect. The model considers the non-linear effects of seasonal and cyclical variations on the probability of foodborne illnesses and analyzes the lagged effects from 0 to 30 days.

According to the cross-lagged correlation (CLC) test, only temperature had a 17-day lag effect among the climate variables. Relative humidity (RH), total precipitation (Precip), road density (density), per capita GDP (GDP), and the number of food and beverage spots in the nearby 1 km range (count) were included in the model.

## 3. Results

### 3.1. Epidemiologic Observations and Trends

From 2014 to 2018, 11,020 cases of foodborne diseases were surveyed in the main urban area of Hangzhou, including 638 cases of *V. parahaemolyticus* infection. In the population structure, the positive detection rate of *V. parahaemolyticus* was 5.65% in the male group and 5.94% in the female group, with a slight female predominance. The detection of *V. parahaemolyticus* varied among age groups: 0.31% in the ≤15-year group, 8.11% in the 16–24-year group, 9.49% in the 25–44-year group, 7.72% in the 45–64-year group, and 4.62% in the ≥65-year group, with infections showing a middle-high and two-low distribution throughout the age structure.

The positive detection rate of *V. parahaemolyticus* varied across occupations. Among the cases with known occupational information, cadres and staff, houseworkers and the unemployed, and migrant workers ranked in the top three, with detection rates of 9.36%, 8.66%, and 5.56%, respectively, while the lowest detection rate of 0.9% was found in students. Regarding food types, the detection rates of *V. parahaemolyticus* were, in descending order, 9.32% in aquatic products, 5.57% in meat products, 3.24% in cereal products, 3.02% in vegetable products, and 1.48% in dairy products.

Further analysis of the places where suspected case-exposed foods were eaten revealed that the detection rate of *V. parahaemolyticus* was higher in the catering services and collective canteens, at 10.39% and 9.77%, respectively, whereas the detection rate of *V. parahaemolyticus* was lower when eating purchased food from a retail market. The detection rate of *V. parahaemolyticus* in the main urban area of Hangzhou showed fluctuating changes on an annual basis during the period from 2014 to 2018, with the highest point in 2016 and the lowest value in 2018, whereas the average annual temperature was the highest in 2016 and the lowest in 2018 in these 5 years. The detection rate of *V. parahaemolyticus* revealed a seasonal distribution, mainly concentrated between May and October, accounting for 99.69% of the total number of cases. In August, a peak was observed, indicating that *V. parahaemolyticus* infections mainly occurred during months with higher temperatures.

**Figure 3:**
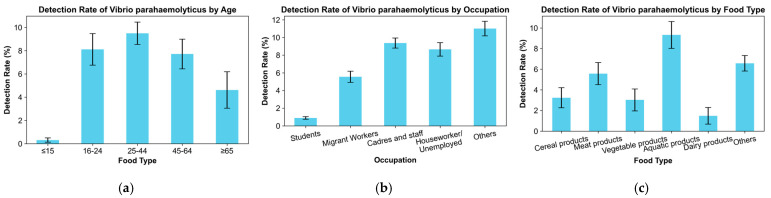

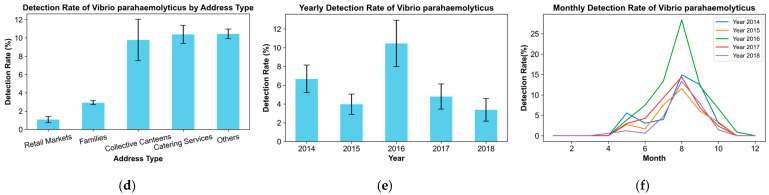
Distribution of the positive detection rate of *V. parahaemolyticus*. (**a**) Detection rates of *V. parahaemolyticus* among age groups; (**b**) Detection rates of *V. parahaemolyticus* among occupations; (**c**) Detection rates of *V. parahaemolyticus* in different exposed foods; (**d**) Detection rates of *V. parahaemolyticus* in different exposed eating places; (**e**) Detection rates of *V. parahaemolyticus* between different years; (**f**) Detection rates of *V. parahaemolyticus* between different months.

In summary, among the foodborne disease surveillance cases that occurred between 2014 and 2018 in the main urban area of Hangzhou, *V. parahaemolyticus* infections differed significantly in terms of population structure, age distribution, occupational structure, and food exposure history (Figure 3). The positive detection rates were similar for males and females, but there were some differences between different age groups and occupational structures. The highest positive detection rates were found in the 25–44-year age group, and the rate for cadres and staff was the highest among occupations. In terms of food exposure history, aquatic products were the food groups with the highest detection rates of *V. parahaemolyticus*, and catering services were the food-eating places with the highest detection rates. *V. parahaemolyticus* infections occurred mainly in months and years with higher temperatures.

**Figure 4:**
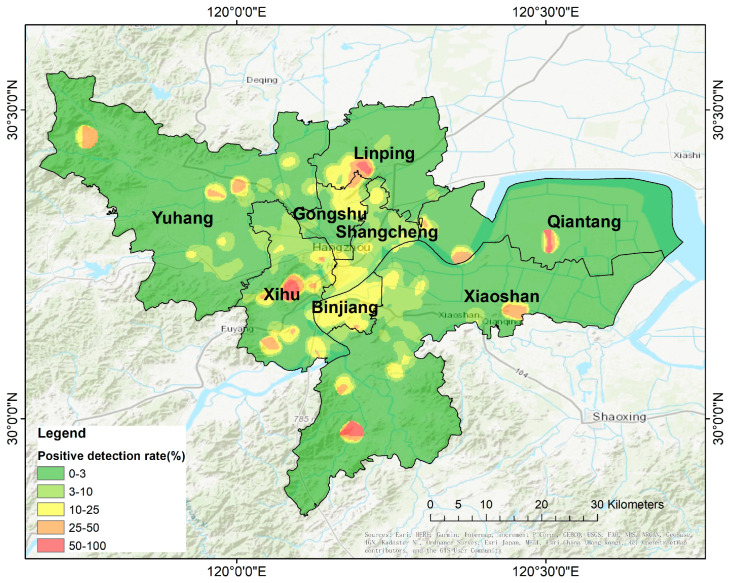
Cumulative kernel density distribution of the *V. parahaemolyticus* detection rate in the main city of Hangzhou, 2014–2018.

According to the geographical kernel density distribution map (Figure 4), the positive rate of *V. parahaemolyticus* showed clear differences in geospatial distribution. Many high kernel density areas have formed on both sides of the Qiantang River, especially in the Xihu, Binjiang, and Gongshu Districts. This proportion gradually decreased with increasing distance from the city center, reflecting the uneven distribution of *V. parahaemolyticus* infection in the city. Therefore, when formulating targeted prevention and control strategies for *V. parahaemolyticus*, it is important to consider geographical distribution characteristics and strengthen the monitoring and management of high-risk areas to ensure public health and food safety.

### 3.2. Analysis of Influencing Factors

A survey of *V. parahaemolyticus* diseases occurring in the main urban area during 2014–2018 showed that the occurrence of the disease was closely related to population characteristics, meteorological factors, and social factors. Some variables were excluded to avoid multicollinearity among the independent variables. For example, there is a significant covariance between the daily mean, maximum, and minimum temperatures, whereas air pressure and solar radiation are highly correlated.

In terms of population characteristics, we found that children younger than 15 years had the lowest risk of infection. Young people aged 16–24 years (odds ratios (OR) = 12.970) had the highest risk of infection, followed by young adults aged 25–44 years (OR = 11.798), middle-aged and older adults aged 45–64 years (OR = 11.134), and older adults older than 65 years (OR = 6.416). In terms of occupation, the highest risk of infection was found among houseworkers and the unemployed (OR = 3.12), followed by migrant workers (OR = 2.514), and cadres and staff (OR = 2.261), while the lowest risk of infection was found among students.

The type of food and place of consumption were also found to affect *V. parahaemolyticus* infection rates. Among the suspected food items, aquatic products were the main source of infection (OR = 1.904), followed by meat products (OR = 1.582) and dairy products (OR = 1.230). Conversely, vegetable products (OR = 0.711) exhibited a relatively lower risk of infection. Among the places where suspected food vehicles that cases were exposed to were prepared, collective canteens (OR = 6.544) were one of the eating places with the highest risk of infection, followed by the catering services (OR = 3.523), which has a relatively high flow of people.

Among the meteorological factors, temperature (OR = 1.196), one of the most significant risk factors, was closely related to the risk of *V. parahaemolyticus* infection, while other meteorological factors also had an impact on infection. Socioeconomic factors were also found to contribute to *V. parahaemolyticus* infections. The level of per capita GDP (OR = 1.029) contributed more to the risk of *V. parahaemolyticus* infection, while the remaining factors contributed less.

According to the results of this study, for the main urban area of Hangzhou, age 16–24 years; occupations including houseworkers and the unemployed, cadres and staff, or migrant workers; eating aquatic and meat products; eating places of collective canteens and catering services; high temperatures; and high per capita GDP were risk factors for *V. parahaemolyticus* infection (Table 1).

### 3.3. Climate Lag Effect

**Figure 5:**
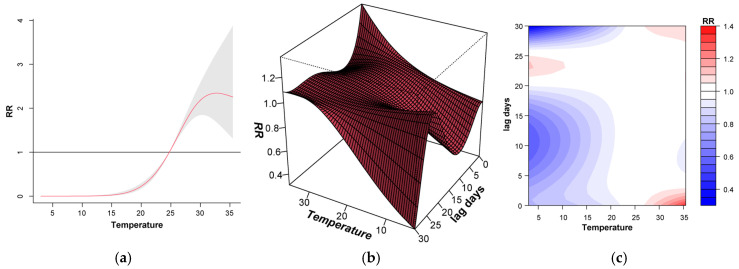
Daily mean temperature and overall exposure response to *V. parahaemolyticus* foodborne illness risk. (**a**) Overall exposure–response relationship graph; (**b**) Relative risk under different temperatures and lags—3D; (**c**) Relative risk under different temperatures and lags—2D. Notes: In Figure 5a, the red line is the mean relative risks. The horizontal line at Relative risk (RR) = 1 is the no-effect baseline. The grey shaded areas are the 95% confidence interval of risk estimates.

Climatic factors had a lagging effect on the risk of *V. parahaemolyticus* infection. Figure 5a shows that the risk of *V. parahaemolyticus* infection increases with the increase in daily mean temperature. When the temperature reaches 35 °C, the risk will be significantly increased (Relative Risk (RR): 2.28; 95% Confidence Interval (95% CI): 1.38–3.76). Figure 5b,c show the relationship between temperature, lag time, and risk value. In the case of high temperature (daily mean temperature exceeding 25 °C), there is a risk of infection when the lag time is 1–4 days, and the risk decreases with the increase in lag time (RR: 1.01; 95% CI: 1.00–1.02). For lower temperatures (daily mean temperature lower than 15 °C), the overall risk value is lower, but there is a risk of infection when the lag is 21–25 days (RR: 0.89; 95% CI: 0.63–1.24).

**Figure 6:**
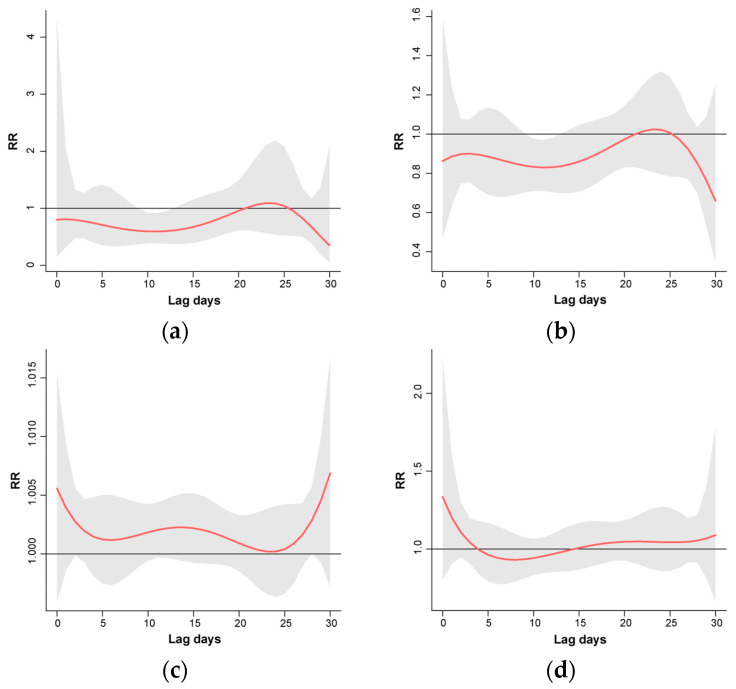
Relative risk and lag effect of *V. parahaemolyticus* infection under different temperature scenarios: (**a**) 5 °C, (**b**) 15 °C, (**c**) 25 °C, and (**d**) 35 °C. Notes: The red line is the mean relative risks. The horizontal line at Relative risk (RR)=1 is the no-effect baseline. The grey shaded areas are the 95% confidence interval of risk estimates.

Figure 6 illustrates the RR of *V. parahaemolyticus* infection across different daily mean temperatures. It demonstrates that at low daily mean temperatures, such as 5 °C, the RR of infection remains comparatively low but slightly increases with a 21-day lag (RR: 0.81; 95% CI: 0.32–2.04). Upon reaching a daily mean temperature of 35 °C, the risk of *V. parahaemolyticus* infection reached the maximum within 1–2 days (RR: 1.18; 95% CI: 0.91–1.52) and then decreased.

**Figure 7:**
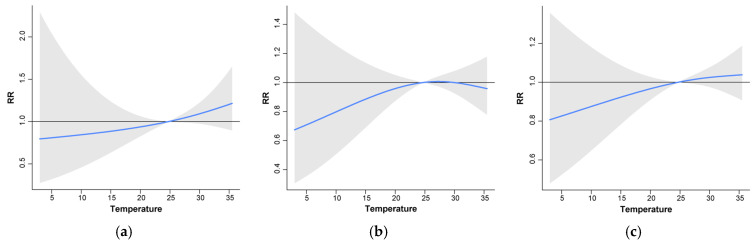

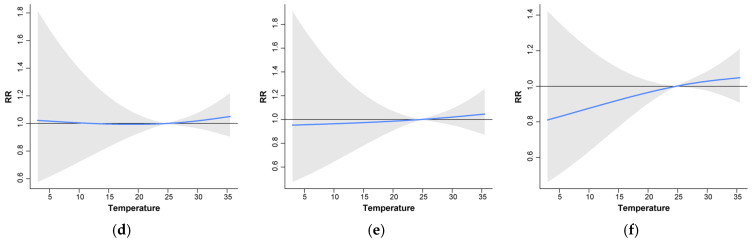
Relative risk of *V. parahaemolyticus* infection and its temperature at different lag days: (**a**) Lag day 1, (**b**) Lag day 5, (**c**) Lag days 18, (**d**) Lag day 21, (**e**) Lag day 26, and (**f**) Lag days 27. Notes: The blue line is the mean relative risks. The horizontal line at Relative risk (RR) = 1 is the no-effect baseline. The grey shaded area are the 95% confidence interval of risk estimates.

Figure 7 complements these findings by exploring the interaction between temperature and lag days, revealing distinct patterns in infection risk across temperature ranges. With a 1-day lag, the risk of *V. parahaemolyticus* infection in high temperatures (21–25 °C) was significantly higher than that in low temperatures. At a 5-day lag, the risks of infection were low across both high and low temperatures; an 18-day lag increased infection risks above 25 °C. A 21-day lag period indicated infection risks existed under both high and low-temperature conditions, while a 26-day lag saw the increased risks under high-temperature conditions but decreased under low-temperature conditions. Generally speaking, there is a risk of infection when the daily mean temperature is lower than 15 °C, while when the daily mean temperature is higher than 25 °C, there is a risk of infection for 1–4 days and 21–30 days. Different infection risk models were observed for the low and high-temperature ranges. Therefore, it is necessary to comprehensively consider the lag effect under different temperature conditions to better predict the modes and trends of disease transmission.

## 4. Discussion

### 4.1. Impact Factors

*V. parahaemolyticus* is often considered one of the main causes of gastroenteritis associated with the consumption of raw or improperly cooked seafood. *V. parahaemolyticus* grows at a minimum temperature of 8.3 °C [15], but is more likely to multiply in warmer environments [52]; therefore, the rate of infection increases in warmer months. The true burden of *V. parahaemolyticus* may be underestimated because many *V. parahaemolyticus* infections occur sporadically. Moreover, *V. parahaemolyticus* mainly causes gastroenteritis, which is usually short and self-limiting, with a high proportion of underreporting [53,54].

Based on the epidemiological statistics of foodborne diseases, the highest detection rate of *V. parahaemolyticus* was found in patients between 25 and 44 years of age (340/3581), which is consistent with the detection rate of *V. parahaemolyticus* in Zhejiang Province [55] and Shanghai Municipality [56]. However, compared to other age groups, those aged 16–24 years showed a higher risk of infection, followed by those aged 25–44 years and those aged 45–64 years. This is mainly due to the fact that multiple logistic regression takes into account a variety of risk factors, including occupation, dietary habits, and the surrounding climatic environment. The 16–24-year group mainly includes students, who tend to live a fast-paced life, eat out frequently, and are more likely to come into contact with venues with a poor food safety environment, which increases the risk of infection. In addition, they may lack sufficient food safety knowledge and experience, pay less attention to food safety and hygiene, and easily overlook potential food contamination risks, thereby posing relatively high risks. This demographic’s higher likelihood of infection is well-documented in studies conducted in southeastern China, aligning with the situation in this region [57,58]. In terms of occupational distribution, the detection rate of *V. parahaemolyticus* was highest among cadres and staff, followed by houseworkers and the unemployed and migrant workers. However, houseworkers and the unemployed populations had the highest risk of infection, followed by migrant and cadre workers. This differs from the higher infection risk observed among migrant workers and laborers in Shanghai [59]. The influence of occupation on *V. parahaemolyticus* infection is mainly attributed to exposure to contaminated food and the level of immunity in different occupational groups. For example, the risk of *V. parahaemolyticus* infection among houseworkers and the unemployed may be related to their daily dietary habits, such as the consumption of insufficiently heated food and improperly stored leftovers [60]. Furthermore, cadres and staff usually eat in the collective canteens of their organization, which may lead to collective foodborne disease outbreaks if the ingredients are not handled properly. Migrant workers who often eat out are easily exposed to catering services with poor hygiene conditions, which increases the risk of infection. It is important to recognize that each demographic group faces varying levels of exposure to *V. parahaemolyticus*, which implies the need for tailored interventions. Consequently, measures such as enhancing food safety protocols, promoting stringent hygiene standards in communal dining settings, and raising awareness among vulnerable populations are crucial for mitigating *V. parahaemolyticus* infections and improving public health outcomes.

In terms of suspected food types, the results of multifactorial analysis showed that the consumption of aquatic products and related products was a significant risk factor for *V. parahaemolyticus* infection. *V. parahaemolyticus*, a halophilic bacterium, tends to proliferate in seafood and is the primary causative agent of seafood-associated gastroenteritis worldwide [61]. The detection rate of *V. parahaemolyticus* in aquatic products in Zhejiang Province was among the highest in the country during 2014–2017 [62]. Furthermore, Hangzhou, a large coastal city in the province with a large consumption of aquatic products, is prone to outbreaks of *V. parahaemolyticus* foodborne disease if food is not handled properly during processing and preparation. Meat and meat products are also risk factors for *V. parahaemolyticus* infection, and many ready-to-eat (RTE) foods do not require further processing before consumption, increasing their likelihood of containing *V. parahaemolyticus*. To reduce the risk of infection, relevant departments should conduct extensive publicity to educate the public about cooking seafood and meat products thoroughly, cooking them for consumption, and reducing raw food consumption. Among the places where suspected food vehicles that cases were exposed to are consumed, collective canteens are considered high-risk places for *V. parahaemolyticus* infections. Poor hygiene conditions are associated with the presence of *V. parahaemolyticus* in food, which leads to outbreaks of foodborne diseases. Indeed, catering services have the highest detection rate of *V. parahaemolyticus* and therefore require enhanced food safety management to reduce the risk of infection. This is in contrast with developing countries, where the main site of infection is the home [63].

Socioeconomic factors such as per capita GDP are considered risk factors affecting *V. parahaemolyticus* infection. In general, areas with low economic levels are prone to *V. parahaemolyticus* foodborne illnesses because of poor sanitary conditions. In contrast, areas with higher economic levels, such as Catalonia, have a relatively lower risk of foodborne disease outbreaks because of easier access to medical care for their residents [64]. However, in areas with more developed economic levels, such as Hangzhou, a high economic level may lead to an increase in the frequency of residents eating out, thus increasing the risk of foodborne disease outbreaks, such as those observed in Michigan in the United States [65] and Semnan Province in Iran [66]. Therefore, it is important for relevant authorities to remind the public to form good dining habits and hygiene practices during public education.

The meteorological risk factors related to *V. parahaemolyticus* infection include relative humidity, daily mean temperature, and total precipitation. Daily mean temperature and relative humidity were found to be positively correlated with *V. parahaemolyticus* infection, whereas precipitation had a weak negative correlation with *V. parahaemolyticus* infection, which is consistent with the results of a study conducted in Taiwan [33]. A high-temperature and humid climate is conducive to the growth and reproduction of microorganisms such as *V. parahaemolyticus*, which can lead to the rapid deterioration of food. The results of this study are consistent with the overall situation in Zhejiang Province [38]. High temperature and humidity can cause the multiplication of *V. parahaemolyticus* and lead to food contamination; therefore, attention should be paid to the safe storage of food.

### 4.2. Temperature Lag

The spread of *V. parahaemolyticus* may be more susceptible to meteorological factors because the bacteria exhibit different survival and transmission characteristics under different temperature and humidity conditions. A previous study showed that all syndromes of *V. parahaemolyticus* infections were more prevalent during warmer months along the Gulf Coast [43]. Another study of *V. parahaemolyticus* in the environment of farmed oysters in Oregon found that the density of *V. parahaemolyticus* in seawater was positively correlated with water temperature, with the highest density of *V. parahaemolyticus* detected in the summer months [67]. The density of *V. parahaemolyticus* in aquatic products was also found to be significantly higher in summer than in winter in northern Chinese cities [68,69]. Several studies have confirmed the effects of temperature on the survival and transmission of *V. parahaemolyticus* [15,35,52]. Under certain conditions, the chance of infection with *V. parahaemolyticus* increases with increasing temperature. In particular, according to the results of multiple logistic regression, temperature is the main risk factor for *V. parahaemolyticus* infection in the main city of Hangzhou in terms of meteorological factors. Therefore, when studying the factors of *V. parahaemolyticus* infection in the main urban area of Hangzhou, we focused on the effect of temperature lag on infection.

In a lower temperature environment (such as below 15 °C), the risk of infection does not increase significantly at the beginning, but with the passage of time, the risk of infection occurs when there is a lag of 21–25 days. In a higher temperature environment (such as above 25 °C), the infection risk is very high in the first 4 days and then decreases with the passage of time, followed by a slight infection risk after a lag of 21 days. This was confirmed in studies in England and Wales, where the peak lag effect between high temperatures and foodborne illness episodes occurred at 1 week but persisted for up to 5 weeks [70], demonstrating the persistent effect of high temperatures on illness episodes. Therefore, it is important for relevant authorities to monitor food safety and take effective measures to ensure public health during hot weather.

## 5. Conclusions

In this study, general statistical descriptive methods, multi-factor logistic regression analysis methods, and the DLNM model were used to comprehensively analyze the factors influencing *V. parahaemolyticus* foodborne illnesses in the main urban area of Hangzhou City in terms of demographic characteristics, socioeconomic factors, and meteorological environment. The results of this study showed that the cases of *V. parahaemolyticus* infection in the main urban area of Hangzhou were related to the age of the patients, their occupation, the type of suspected exposed food, the place where the suspected exposed food was eaten, daily mean temperature, relative humidity, wind speed, per capita GDP, and other factors. The lag effect of temperature variables on *V. parahaemolyticus* infection was investigated using the DLNM model, and the risk of *V. parahaemolyticus* infection showed different exposure trends under different temperature conditions. In a low-temperature environment, the infection risk significantly increased after a lag of 21 days, whereas in a high-temperature environment, the infection risk reached the highest level on the same day, with two risk periods of 1–4 and 21 days lag.

This study was conducted on a daily scale, revealing the lag effect at a subtle daily scale compared to previous interannual, monthly, or weekly scale studies. These findings provide guidance and support for the relevant government departments in Hangzhou to prevent and control foodborne diseases. Future research should seek to further explore the perspectives of environmental factors, population behavioral habits, and medical and health conditions and formulate more effective prevention and control strategies to reduce the incidence of *V. parahaemolyticus* infection in the main urban area of Hangzhou. However, this study has two main limitations. Firstly, there were problems with missing data for some key variables, such as the presence of many other statistically unknown data for eating places and suspected exposed food data, which may have led to results that do not fully reflect the factors influencing *V. parahaemolyticus* infection in the main urban area of Hangzhou. Secondly, the analysis of social factors failed to include factors such as personal hygiene habits and educational level. Future studies should incorporate additional social factors to fully elucidate the mechanisms underlying infection risk.

## Figures and Tables

**Table 1:** Multiple logistic regression analysis of factors influencing *V. parahaemolyticus* infection.

Variable		β	S.E	Waldχ^2^	*p*	OR
Age	≤15			57.301	<0.001 ***	Reference
	16~24	2.563	0.365	49.337	<0.001 ***	12.970
	25~44	2.468	0.384	41.222	<0.001 ***	11.798
	45~64	2.410	0.396	37.062	<0.001 ***	11.134
	≥65	1.859	0.436	18.193	<0.001 ***	6.416
Careers	Students			27.728	<0.001 ***	Reference
	Migrant workers	0.922	0.263	12.261	<0.001 ***	2.514
	Cadres and staff	0.816	0.236	11.905	<0.001 ***	2.261
	Houseworkers and the unemployed	1.138	0.262	18.865	<0.001 ***	3.120
	Others	1.147	0.241	22.612	<0.001 ***	3.149
Suspected exposure food groups	Cereal products			30.640	<0.001 ***	Reference
	Meat products	0.459	0.205	5.005	0.025 *	1.582
	Vegetable products	–0.341	0.259	1.733	0.188	0.711
	Aquatic products	0.644	0.193	11.079	<0.001 ***	1.904
	Dairy products	0.207	0.349	0.351	0.553	1.230
	Others	0.559	0.186	9.042	0.003 **	1.749
Suspected exposed food-eating places	Retail markets			154.437	<0.001***	Reference
	Home	0.582	0.335	3.008	0.083	1.789
	Collective canteens	1.879	0.434	18.721	<0.001 ***	6.544
	Catering services	1.259	0.347	13.165	<0.001 ***	3.523
	Others	1.818	0.332	29.997	<0.001 ***	6.162
Socioeconomic factors	Road density (km/km^2^)	0.0004	0.0001	37.345	<0.001 ***	1.000
	Per capita GDP	0.029	0.009	9.351	0.002 **	1.029
	Number of food and beverage outlets	0.0003	0.0002	3.738	0.053	1.000
Meteorological factors	Relative humidity (%)	0.020	0.005	17.553	<0.001 ***	1.020
	Daily mean temperature (°C)	0.179	0.012	237.099	<0.001 ***	1.196
	Total precipitation (mm)	–0.011	0.006	4.004	0.045 *	0.989

* *p* < 0.05; ** *p* < 0.01; *** *p* < 0.001.

## Data Availability

The data presented in this study are available on request from the corresponding author due to ethical restrictions regarding participant privacy and confidentiality.
